# Longitudinal Assessment of Sexual Behavior and Relationship Quality During the First Year of the COVID-19 Pandemic in Britain: Findings from a Longitudinal Population Survey (Natsal-COVID)

**DOI:** 10.1080/00224499.2024.2432000

**Published:** 2025-01-15

**Authors:** Naomi Miall, Alice Aveline, Andrew J. Copas, Raquel Bosó Pérez, Andrew J. Baxter, Julie Riddell, Laura Oakley, Dee Menezes, Anne Conolly, Chris Bonell, Pam Sonnenberg, Catherine H. Mercer, Nigel Field, Kirstin R. Mitchell, Malachi Willis

**Affiliations:** a MRC/CSO Social and Public Health Sciences Unit, University of Glasgow; b School of Public Health, Imperial College London; c Institute for Global Health, University College London; d Faculty of Epidemiology and Public Health, London School of Hygiene & Tropical Medicine; e Institute for Health Informatics, University College London; f NatCen Social Research; g Faculty of Public Health and Policy, London School of Hygiene & Tropical Medicine

## Abstract

While the impact of social restrictions on sexual and romantic life early in the COVID-19 pandemic has been widely studied, little is known about impacts beyond the initial months. We analyzed responses from 2,098 British adults (aged 18–59) taking part in the Natsal-COVID study (Waves 1 and 2). Participants were recruited via a web panel and surveyed twice: four months and one year after the start of the UK’s first national lockdown (July 2020 and March 2021). Changes in the prevalence and frequency of participants’ physical and virtual sexual behaviors between the two surveys were analyzed using multinomial logistic regression. Changes in the quality of intimate relationships were modeled using logistic regression for the 1,407 participants in steady relationships, adjusting for age, gender, and relationship status. The reported prevalence of any sexual activity amongst the full sample increased over the study period (from 88.1% to 91.5%, aOR = 1.50, 95% CI 1.23–1.84). Increases were observed for physical (aOR = 1.41, 95% CI 1.15–1.74) and virtual (aOR = 1.20, 95% CI 1.07–1.34) activities, particularly masturbation (aOR 1.53, 95% CI 1.37–1.72). Increases were larger for men than women. The proportion of participants in steady relationships whose relationship scored as “lower quality” increased (from 23.9% to 26.9%, aOR = 1.28, 95% CI 1.10–1.49). These findings have implications for understanding sexual health needs during disasters and planning sexual health service priorities following the pandemic.

## Introduction

**Figure 1.**
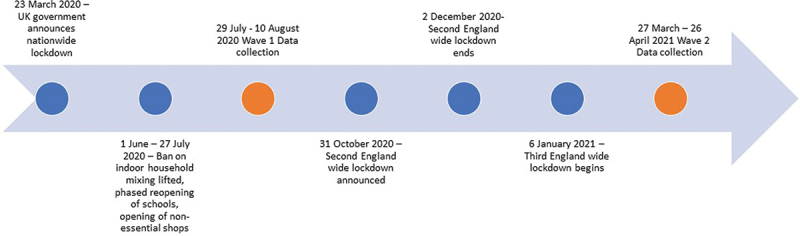
Timeline of UK COVID restrictions and NATSAL-COVID data collection.

In response to the COVID-19 pandemic, declared by the WHO in March 2020, governments around the world introduced restrictions on social interaction. In Britain, stay-at-home and social-distancing policies were mandated with varying levels of stringency over time, with the intention to reduce transmission of the SARS-COV-2 virus (Figure 1). These social restrictions had wide-ranging and diverse consequences for all aspects of life, including sexual and relational wellbeing (Cascalheira et al., 2021; Chow et al., 2021; Hensel et al., 2023; Mann et al., 2023; Mercer et al., 2022). Previous studies have established associations between sexual behavior and aspects of sexual health such as sexually transmitted infections and unplanned pregnancy (Cao et al., 2015; Tsai et al., 2019). Therefore, exploring the evolution of sexual behavior over the course of the pandemic not only improves our understanding of sexual behavior during largescale social disruptions, but also provides evidence to inform sexual and reproductive health planning and priorities post- lockdown. Furthermore, there are well-established associations between sexual behavior and other important health outcomes, including cardiovascular health, immune system functioning, stress reduction, self-esteem, and overall quality of life (Flynn et al., 2016; Pietromonaco & Collins, 2017; Robles et al., 2014). It follows that understanding the impact of the pandemic on changes in sexual behavior and romantic relationships has important implications for people’s overall health.

At the start of the pandemic, many cross-sectional studies were conducted to examine the immediate impact of the COVID-19 pandemic on sexual behavior, with most reporting declines in partnered sexual behavior or overall sexual well-being (Delcea et al., 2021; Firkey et al., 2022; McKay et al., 2023; Sanchez et al., 2020; Wignall et al., 2021). Other studies reported increases in solo sexual behaviors, including masturbation and online porn use, in the early months of the pandemic due to greater time at home or online (Cascalheira et al., 2021; Lau et al., 2021; Melca et al., 2021; Zattoni et al., 2020). However, the cross-sectional design of these studies limited understanding of changes in sexual behavior and well-being over the course of the pandemic since participants could only provide data on behavior at a single point in time. In addition to the limitations of the cross-sectional design, some studies did not account for potentially important effect modifiers, for example, whether participants had a co-habiting partner, which determined whether individuals were permitted to physically meet their partner under stay-at-home measures (Delcea et al., 2021; Lau et al., 2021; Sanchez et al., 2020; Zattoni et al., 2020).

Studies of relationship satisfaction in the early months of lockdown have suggested a complex picture. Some have suggested that the pandemic was associated with declines in relationship quality. For example, Luetke et al. (2020) found that marital conflict levels rose among a nationally representative sample of US adults in the first month of the pandemic and Schmid et al. (2021) found that more respondents reported decreases in relationship satisfaction than increases over the first three months of lockdown in Germany. Furthermore, greater levels of perceived anxiety in adults were associated with increased relationship instability in the first six months of the pandemic (Balzarini et al., 2022; Ogan et al., 2021; Rodríguez-Domínguez et al., 2022). Other studies reported more positive findings. Our Natsal-COVID Wave 1 study was the first wave of a nationally representative study of sex and relationships in Britain during the pandemic, conducted four months after the first UK lockdown began. The findings suggest that 40% of British participants in steady relationships reported changes in relationship quality in the initial pandemic months, with perceived improvement more common than perceived decline (Mitchell et al., 2022). The different conclusions of these studies may reflect variation in the way that relationship quality is conceptualized and measured, or country level differences in the impacts of restrictions.

The imposition of social restrictions by governments did not impact everyone equally. Opportunities for partnered sexual activity became dictated by living arrangements, with particularly adverse consequences for those who were not in cohabiting relationships, a living situation especially common among young people (Long et al., 2021; McKinlay et al., 2022). Among heterosexual US college students, over 50% reported decreased opportunities to have sex and decreased frequency of partnered sex (Firkey et al., 2022). Results from the Natsal-COVID Wave 1 study found that, 50% of British participants perceived no change in the frequency of partnered sex in the four months post-lockdown, compared with the months pre-lockdown (Mercer et al., 2022). However, Mercer et al. found that those in casual or non-cohabiting relationships reported less frequent partnered sex and more frequent masturbation, sex toy use and virtual activities. A large online, nationally representative study of US adults also reported decreased partnered sexual behaviors and increased solo masturbation amongst those who did not cohabit with a partner or were single (Hensel et al., 2023). This observed shift from in-person, partnered activities to virtual activities amongst those in less formal relationships may have negative health implications. Rosenberg et al. (2021) found that remote sexual connections (e.g., internet sex, dating apps) did not substitute for the positive mental health benefits of in-person sexual connections among a sample of US adults.

In addition to the observed differences as a result of cohabitation status, there is also evidence that changes in sexual behavior and relationship satisfaction, which occurred in the early months of the pandemic, differed by gender. The Natsal-COVID Wave 1 study found that among those in steady relationships in Britain, a larger proportion of women than men reported a reduction in their interest in sex, compared to the period before the pandemic, although gender was not associated with changes in overall perceived relationship quality (Mitchell et al., 2022). Among a convenience sample of Italian health care workers and acquaintances, women reported lower sexual desire than men (De Rose et al., 2021). A meta-analysis examining pandemic changes in women’s sexual function suggested that women had more problems with arousal, orgasm, satisfaction and pain during the pandemic compared to before the pandemic; however, no differences in frequency of intercourse were observed (Hessami et al., 2022).

The existing evidence base detailing the impact of the pandemic on sexual behavior and relationship quality is almost entirely focused on the early months of the pandemic. There is very little research on the effect of pandemic restrictions on sexual behavior and romantic relationships after autumn 2020, and existing longitudinal studies exploring changes over the course of the pandemic rely on non-representative convenience samples (Mann et al., 2023; Storer et al., 2023; Tan, 2022). Thus far, evidence exploring whether the observed early changes to sexual behavior and romantic relationships were sustained beyond the initial months of restrictions for most adults has been limited.

### Present Study

We used the Natsal-COVID study, a nationally representative, non-probability sample of the British population to examine changes in sexual behavior and levels of relationship satisfaction in the first year after the start of the initial British COVID-19 lockdown. We investigated whether changes observed in the first few months of the pandemic persisted one year after the beginning of restrictions or were transient consequences of the pandemic’s initial disruption. We also examined whether inequalities in the impact of the pandemic on sexual behavior and relationship quality, identified in the early stages of the crisis, persisted as social restrictions evolved. The longitudinal nature of the sample, nested within a repeat cross-sectional study, addresses an important gap in the existing evidence and allows us to examine how sexual behavior and relationship satisfaction changed for the same large, nationally quasi-representative group of participants over a year.

RQ1: How did the prevalence of specific sexual behaviors change between the initial national lockdown period (i.e., first four months) and one year after the initial national lockdown began? Did any observed changes in prevalence vary by age, gender identity, relationship status or cohabitation status?

RQ2: How did the frequency of specific sexual behaviors change between the initial lockdown period and one year after the initial lockdown?

RQ3: Did participants in steady relationships experience changes in relationship quality between the initial lockdown period and one year after the initial lockdown? Did any changes in relationship quality vary by age, gender identity or cohabitation status?

## Method

### Study Design

The Natsal-COVID study aimed to examine the impact of the COVID-19 pandemic and associated restrictions on the sexual health, sexual behavior and romantic relationships of British adults, using two web panel survey waves, administered by Ipsos. The first wave (Natsal COVID Wave 1, hereafter called Wave 1) was administered between 29 July and 10 August 2020, four months after the first UK national lockdown began on March 26 2020 (Figure 1). In the four months prior to the survey, participants had faced stringent stay at home orders between March and June, with phased restriction easing during July. By the end of July, non-essential shops, pubs, restaurants and hairdressers were able to re-open, and groups including two households were able to gather indoors, or groups of up to six outdoors (Institute for Government, 2022). The second wave of data collection (Natsal-COVID Wave 2, hereafter called Wave 2) ran between 27 March and 26 April 2021, one year after the first national lockdown began. The four-month period prior to Wave 2 overlaps with the third national lockdown (starting January 6 2021, with phased easing from March 2021; Institute for Government, 2022). At the time of Wave 2 data collection, schools had recently re-opened following a four-month closure, and socializing outside of the household was restricted to small outdoor gatherings. Non-essential retail and outdoor pubs and restaurants were able to re-open midway through this second data collection wave. Participants completed the online survey on personal devices and provided consent via an online form (available at https://www.natsal.ac.uk/natsal-covid-study). Detailed methods are published elsewhere (Dema et al., 2021, 2022).

### Sample

British residents aged 18–59 were eligible for inclusion in the Wave 1 sample, which was selected using quota-based sampling and contacted by e-mail. Quotas were defined for gender, age, geographic region and social grade. In total, 6,654 participants completed Wave 1, and 6,658 participants completed Wave 2. The nested longitudinal sample comprised 2,098 people who participated in both Wave 1 and Wave 2. The sample was weighted using a longitudinal weight, based on gender, age, ethnicity, region, social grade and sexual orientation, to account for sample design and attrition. Participants’ sociodemographic characteristics, sexual behavior, and relationship quality in the longitudinal sample were compared with the complete Wave 1 sample to evaluate how well the survey weights accounted for attrition (Dema et al., 2022).

Of the 2,098 participants that took part in both Waves 1 and 2, after weighting, 50.4% identified as women, 85.8% as white and 60.8% were in steady cohabiting relationships at Wave 1 (Table 1). This nested longitudinal sample was broadly similar in these characteristics to the Wave 1 cross-sectional sample (*n* = 6,654), although the longitudinal sample was older, as detailed in Dema et al. (2022).

**Table 1.** Weighted sociodemographic characteristics of the Natsal-COVID longitudinal sample (n = 2,098).

	Natsal-COVID Wave 1^a^ % (95% CI)	Natsal-COVID Wave 2^a^ % (95% CI)
**Age group**^b^		
18–24	11.0 (8.8–13.7)	8.4 (6.6–10.6)
25–29	19.0 (16.6–21.6)	19.1 (16.5–22.0)
30–39	24.1 (21.8–26.6)	24.5 (22.1–26.9)
40–49	23.0 (21.1–25.2)	23.2 (21.2–25.3)
50–59	22.8 (21.0–24.8)	24.9 (22.9–26.9)
**Gender**^c^		
Women (including trans women)	50.4 (47.6–53.3)	50.0 (47.2–52.8)
Men (including trans men)	49.4 (46.6–52.2)	49.7 (46.9–52.5)
In another way	0.2 (<0.1–0.7)	0.4 (0.1–1.1)
**Relationship status**		
Steady and cohabiting	60.8 (57.9–63.6)	60.8 (58.0–63.6)
Steady and not cohabiting	6.0 (4.9–7.5)	7.0 (5.6–8.6)
Single	28.6 (26.0–31.4)	26.7 (24.2–29.3)
Other relationship status	4.6 (3.5–5.9)	5.5 (4.0–7.6)
**Ethnicity**		
White	85.8 (83.1–88.1)	85.8 (83.1–88.1)
Mixed/Multiple	2.0 (1.3–2.9)	1.7 (1.1–2.5)
Asian/Asian British	8.1 (6.3–10.4)	8.1 (6.3–10.4)
Black/African/Caribbean	3.3 (2.2–4.8)	3.4 (2.3–5.0)
Other	0.9 (0.4–2.1)	1.1 (0.5–2.4)
**Household**		
Living with children < 18	31.4 (28.9–34.1)	30.6 (28.1–33.2)
**Social grade**^d^		
Upper middle/Middle class	20.7 (18.7–22.9)	22.6 (20.5–24.7)
Lower middle/Skilled working class	53.8 (51.0–56.6)	52.9 (50.1–55.6)
Working class/Lower subsistence	25.4 (22.9–28.1)	24.6 (22.3–27.0)

^a^Natsal-COVID Wave 1 data collection was conducted four months after the first UK national lockdown began. Natsal-COVID Wave 2 data collection was conducted twelve months after the first UK national lockdown began.

^b^Differences in proportions between Wave 1 and Wave 2 reflect that the cohort of 2,098 participants had aged by 8 months. Other differences reflect small differences in the way in which people reported their characteristics at Wave 1 and 2.

^c^Under the Natsal-COVID approach to gender, data reported for men includes trans men and data reported for women includes trans women. Participants identifying their gender “in another way” are excluded from analyses stratified by gender, given the small sample size, but are included in analyses that are not stratified by gender.

^d ^Social grade defined using the categorization from ONS 2011 Census (https://www.ons.gov.uk/census/2011census).

Few participants were in non-cohabiting steady relationships at both survey waves (*n* = 77). We therefore explored changes in sexual behavior among this group (RQ1) cross-sectionally, using the participants in non-cohabiting steady relationships Wave 1 (*n* = 517) and comparing to this group in Wave 2 (*n* = 566), irrespective of whether participants completed both survey rounds.

Natsal-COVID takes an inclusive approach to gender, with data presented for men including trans men and women including trans women. Three participants who identified “in another way” are included in whole sample analyses but are not reported in analyses that compared changes over time between genders given the small sample size.

For the analysis of changes in relationship quality (RQ3), we restricted the sample to participants who were married, in a civil-partnership or self-identified as in a steady relationship at both timepoints (*n* = 1,407). Participants who identified as single, in new, casual, ending or multiple relationships or another relationship status at either timepoint were not included.

### Measures

The Wave 1 questionnaire was designed to measure perceived changes in sexual lifestyles before and after the start of lockdown, without pre-pandemic baseline data. Therefore, the majority of items measured current behavior in relation to recalled previous behavior (i.e., perceived changes) and were not suitable for longitudinal analysis. In contrast, questions about recent sexual behavior and relationship quality could be harmonized between Wave 1 and Wave 2, allowing direct comparison of these variables. For this reason, these variables were the focus of the present study.

#### Sexual Behavior

In both Natsal-COVID survey waves, participants were asked to report the frequency of nine sexual behaviors during the last four months. There were four physical behaviors: a composite measure which combined any vaginal, anal, or oral [VAO] sex, plus separate measures for “other contact with someone’s genital area,” “masturbating,” and “using sex toys (by yourself or with someone else).” There were five virtual behaviors: “messaging via dating apps/online,” “sexting (images or recorded videos),” “using video or voice calls to interact with someone sexually,” “looking at pornography,” and “paying for online sexual services [e.g., live streaming].” Response options for each behavior were collapsed into three frequency categories: “did not do this,” “did this less than weekly” or “did this at least weekly” (Mercer et al., 2022). “Did not do this” was treated as the reference group because this was the most common response selected for all behaviors.

Responses to these nine items were used to derive five aggregate variables reflecting whether the participant had engaged in: any sexual activity (at least one of the nine behaviors); any physical activity; any virtual activity; any partnered sex (VAO sex or other contact with someone’s genital area); and any virtual activity excluding pornography use. These aggregate variables were coded as missing for participants who had missing responses for one or more of the relevant behaviors and who had not engaged with any of the behaviors for which they had complete data. In a sensitivity analysis, only those who were missing data on all relevant sexual behavior items were coded as missing; those who had a mixture of missing responses and “did not do this” responses were coded as “did not do this” for the aggregate variable.

#### Relationship Quality

In the Natsal-COVID survey Waves 1 and 2, participants responded to six items that assessed relationship quality: “I feel supported by my partner,” “My partner and I regularly argue,” “I feel a strong connection with my partner,” “I can confide in my partner about virtually anything,” “I am worried that our relationship might end,” and “I feel happy with my relationship.” These items demonstrated good internal consistency (Cronbach’s α > 0.8). Response options were scored 1 to 5 (1 for not at all true and 5 for completely true; negative statements reverse coded) and each score was multiplied by the factor loading for that item (see Mitchell et al. (2022) and Supplementary 2 for factor loadings). The weighted responses were summed to give an overall score of relationship quality. Histograms showing the distribution of weighted relationship quality scores in Wave 1 were visually inspected to identify cutoffs. For relationship quality, the observed cutoff of 17 corresponded approximately to the 25^th^ percentile. As a result, a weighted score for relationship quality below < 17 was categorized as “lower quality” and a score greater than or equal to 17 was categorized as “higher quality.” Participants with a missing response for any of the six component items were defined as missing for the relationship quality score.

### Analysis

To assess whether the prevalence of sexual behaviors changed from the initial lockdown period to one year later (RQ1), the relative odds of reporting having engaged in each behavior at least once in Wave 2, compared with Wave 1, were estimated using logistic regression models. In these models, we controlled for age (continuous), gender identity (men, women, in another way) and relationship status (in a steady relationship, not in a steady relationship), each measured at baseline (Wave 1). The models included robust standard errors to account for repeated responses from each participant. To examine the potential moderators of interest (age categorized as 18–34 years or 35–59 years, gender identity, and relationship status, each measured at baseline), we included interaction terms between the survey and each moderating variable. Multinomial logistic regression models were used to assess changes in the frequency of each sexual behavior between the initial lockdown period and one year later. Relative risk ratios of engaging in each behavior infrequently (less than weekly) versus not at all and frequently (at least weekly) versus not at all in Wave 2, compared with Wave 1, were reported. For context, the prevalence of each sexual behavior amongst all participants in steady, non-cohabiting relationships in the cross-sectional sample at each wave is reported, weighted using cross-sectional weights.

A logistic regression model, with robust standard errors to correct for repeated responses from each participant, was used to examine whether the proportion of participants reporting “lower quality” relationships changed between Wave 1 and Wave 2 (RQ3). This model included terms to control for age, gender and cohabitation status at Wave 1. Interaction terms were fitted for each variable to assess whether changes in relationship quality differed between sub-groups. The distribution of response options to each individual component of the aggregate relationship quality score was compared between Waves 1 and 2.

## Results

### Changes in the Prevalence and Frequency of Sexual Behavior (RQ1 & 2)

A higher proportion of participants reported engaging in any sexual activity (i.e., physical or virtual) in the previous four months at Wave 2 (91.5%) than at Wave 1 (88.1%). After adjusting for age, sex and relationship status, the adjusted odds of reporting any sexual activity were 1.50 times higher at Wave 2 compared to Wave 1 (95% CI 1.23–1.84). The adjusted odds of reporting any physical sexual activity (aOR = 1.41 (95% CI, 1.15–1.74) and any virtual sexual activity (aOR = 1.20 (95% CI, 1.07–1.34) were also higher at Wave 2. The increased prevalence of physical sexual activity was largely driven by increases in reported masturbation, sex toy use, and genital contact other than VAO sex. The increased prevalence of virtual behaviors was driven by reported pornography use (Table 2).

**Table 2.** Prevalence of sexual behaviors in previous four months, Natsal-COVID wave 1 versus Natsal-COVID wave 2.

Sexual Behavior	Wave 1%)	Wave 2%)	aOR	*p*
**Any sexual activity**	88.1	91.5	1.50 (1.23–1.84)	<.001
Any physical sexual activity†	86.3	89.6	1.41 (1.15–1.74)	.001
Any partnered sex‡	63.8	65.0	1.07 (0.92–1.24)	.396
Vaginal, oral, and/or anal sex	61.9	63.2	1.06 (0.91–1.22)	.454
Other genital contact	53.7	57.9	1.28 (1.12–1.47)	<.001
Masturbation	57.9	67.0	1.53 (1.37–1.72)	<.001
Using sex toys	22.9	28.1	1.33 (1.19–1.49)	<.001
**Any virtual sexual activity**¶	50.9	54.8	1.20 (1.07–1.34)	.001
Any excluding pornography use**	26.7	26.0	0.96 (0.84–1.10)	.585
Looking at pornography	43.3	46.8	1.19 (1.04–1.36)	.011
Messaging via dating apps/online	20.9	19.1	0.88 (0.75–1.03)	.108
Sexting	15.7	15.5	0.98 (0.83–1.16)	.836
Using video or voice calls	11.5	12.4	1.11 (0.92–1.34)	.277
Paying for online sexual services	6.8	6.3	0.83 (0.58–1.20)	.333

aOR = odds ratio adjusted for age, gender and relationship status at Wave 1 (with 95% CI in parentheses).

†Reported at least one of the following activities in the previous four months: vaginal, anal or oral sex, other contact with someone’s genital area, masturbating, using sex toys (by yourself or with someone else).

‡Reported at least one of the following partnered activities in the previous four months: vaginal, anal or oral sex, other contact with someone’s genital area.

¶Reported at least one of the following in the previous four months: messaging via dating apps/online, sexting (images or recorded videos), using video or voice calls to interact with someone sexually, looking at pornography, paying for online sexual services (e.g., live streaming).

**Reported at least one of the following in the previous four months: Messaging via dating apps/online, sexting (images or recorded videos), using video or voice calls to interact with someone sexually, paying for online sexual services (e.g., live streaming).

We were also interested in how often participants engaged in the different sexual behaviors and how this changed. For most behaviors, the proportion of participants who reported a behavior less than once a week (i.e., infrequently) relative to the proportion not reporting the behavior increased from Wave 1 to Wave 2 (Table 3). However, the relative proportion of participants who reported a behavior at least once a week (i.e., frequently) generally decreased or remained steady from Wave 1 to Wave 2. An exception to this pattern was noted for masturbation; compared to the proportion reporting no recent masturbation, the proportion of participants reporting masturbating either infrequently (aRRR = 1.83, 95% CI 1.57–2.15) or frequently (aRRR = 1.32, 95% CI 1.15–1.50) increased from Wave 1 to Wave 2. There was also a slight increase in the adjusted odds of reported genital contact other than VAO sex frequently (aRRR = 1.12, 95% CI 0.95–1.32) as well as infrequently (aRRR = 1.50 (1.26–1.79)) from Wave 1 to Wave 2, after adjusting for age, gender and relationship status.

**Table 3.** Frequency of sexual behaviors and adjusted relative risk ratio for changes in sexual frequency in the previous four months from Natsal-COVID wave 1 to Natsal-COVID wave 2.

Sexual Behavior Frequency	Wave 1%)	Wave 2%)	aRRR†	*p*
**Vaginal, oral, and/or anal sex**				
Not at all	38.1	36.8	1.00	—
Less than weekly	27.0	30.8	1.17 (0.98–1.40)	.085
At least once a week	35.0	32.4	0.95 (0.80–1.13)	.563
**Other genital contact**				
Not at all	46.3	42.1	1.00	—
Less than weekly	21.2	27.3	1.50 (1.26–1.79)	<.001
At least once a week	32.5	30.6	1.12 (0.95–1.32)	.179
**Masturbation**				
Not at all	42.1	33.0	1.00	—
Less than weekly	20.8	29.7	1.83 (1.57–2.15)	<.001
At least once a week	37.0	37.3	1.32 (1.15–1.50)	<.001
**Using sex toys**				
Not at all	77.1	71.9	1.00	—
Less than weekly	12.1	18.4	1.62 (1.36–1.93)	<.001
At least once a week	10.9	9.7	1.00 (0.82–1.21)	.965
**Looking at pornography**				
Not at all	79.1	80.9	1.00	—
Less than weekly	6.0	8.0	1.37 (1.15–1.64)	.001
At least once a week	14.9	11.2	1.06 (0.89–1.26)	.519
**Messaging via dating apps/online**				
Not at all	84.3	84.5	1.00	—
Less than weekly	8.1	10.0	1.20 (0.83–1.74)	.341
At least once a week	7.6	5.6	0.75 (0.60–0.94)	.013
**Sexting**				
Not at all	88.5	87.6	1.00	—
Less than weekly	5.8	8.4	1.30 (0.97–1.73)	.076
At least once a week	5.7	4.0	0.67 (0.47–0.96)	.027
**Using video or voice calls**				
Not at all	56.7	53.3	1.00	—
Less than weekly	16.1	20.3	1.42 (1.02–1.99)	.039
At least once a week	27.1	26.5	0.78 (0.52–1.19)	.249
**Paying for online sexual services**				
Not at all	93.2	93.7	1.00	—
Less than weekly	2.4	3.9	1.50 (0.99–2.28)	.057
At least once a week	4.4	2.3	0.49 (0.26–0.90)	.021

aRRR = relative risk ratio adjusted for age, gender and relationship status at Wave 1 (with 95% CI in parentheses).

Gender modified the change in prevalence between Wave 1 and Wave 2 of engaging in at least one sexual behavior (interaction term *p* = .031). A substantial increase in reported prevalence over time among men was found for both physical sexual behaviors (aOR = 2.20, 95% CI 1.35–3.58) and virtual sexual behaviors (aOR = 1.34, 95% CI 1.13–1.59), whereas women reported only marginal increases in prevalence (physical behaviors aOR = 1.16, 95% CI 0.94–1.44; virtual behaviors aOR = 1.08, 95% CI 0.93–1.25). At the level of individual behaviors, both men and women reported increases in the prevalence of genital contact other than VAO, sex toy use, masturbation and (marginally) pornography use (Supplementary Table S1).

The increased prevalence of sexual activity in Wave 2 compared with Wave 1 was broadly similar between older and younger participants (aOR for any sexual activity for ages 17–34 = 1.45, 95% CI 0.86–2.47; aOR for ages 35–59 = 1.51,95% CI 1.24–1.83; interaction term *p* = .900) (Supplementary Table S2).

In general, those who were not in steady relationships at Wave 1 reported somewhat greater increases in sexual activity between Waves 1 and 2 than those in steady relationships, although there was no evidence that these estimates differed substantially (aOR for any sexual activity for those in steady relationships = 1.34, 95% CI 0.99–1.81; aOR for those not in steady relationships = 1.66, 95% CI 1.28–2.17; interaction term *p* = .289) (Supplementary Table S3).

The proportion of participants in non-cohabiting, steady relationships who reported any sexual activity did not differ between Wave 1 (95.0%) and Wave 2 (96.0%) (Supplementary Table 4). However, the proportion reporting solo sexual behaviors in this group did increase, including masturbation (65.9% in Wave 1 compared with 75.4% in Wave 2) and looking at pornography (47.2% in Wave 1 compared with 55.8% Wave 2).

Data on sexual behavior was missing at Wave 1 or Wave 2 for 8.0% of participants, with item missingness slightly higher in Wave 2 than Wave 1. Compared with participants with no missing data on the any sexual activity item, participants with missing data were more likely to be older, women, single, not living with children and from an Asian or Asian British background (Supplementary Table S5). In a sensitivity analysis where participants were coded as missing only if they did not have any responses for the relevant variables (those with mixed missing and negative responses were coded as “did not do this”), results were similar although effect sizes were smaller (Supplementary Table S6).

### Changes in Relationship Quality (RQ3)

A total of 1,407 participants were in steady relationships at Wave 1 and Wave 2. The proportion of participants with relationship quality scores categorized as “lower quality” increased between Wave 1 and Wave 2 from 23.9% to 26.9% (aOR = 1.28, 95% CI 1.10–1.49). A deterioration was observed in every component of the relationship quality score between Waves 1 and 2. This change was broadly similar for men and women, younger and older participants, and cohabiting and non-cohabiting participants. However, these averages mask variation at the individual level. Of participants with complete relationship quality data, 9.7% moved from a categorization of “higher quality” to “lower quality” relationships, and 6.6% moved from “lower quality” to “higher quality” relationships.

## Discussion

This study examined how sexual behavior and relationship quality in the general population changed over the first year of the pandemic, comparing the first four months of pandemic restrictions (April – July 2020) to a four-month period which ended one year after the start of lockdowns (December 2020–March 2021). While previous longitudinal studies exploring how sexual behavior changed over the course of the pandemic have examined convenience samples of specific population sub-groups such as men who have sex with men (Mann et al., 2023; Storer et al., 2023), our study is unique in providing evidence of changes in sexual behavior over the first year of the pandemic in a representative general population sample.

There was a slight increase in the prevalence of reported sexual activity over the study period (88.1% at survey Wave 1 vs 91.5% at survey Wave 2), which was observed for physical and virtual activities, and was particularly marked for masturbation. For most behaviors studied, the proportion of participants engaging in the behavior infrequently (less than once per week) increased between Wave 1 and Wave 2, while the proportion of participants who engaged in the behavior frequently (more often than once per week) did not increase. Masturbation was an exception, where the proportion of participants masturbating frequently or masturbating infrequently both increased between Waves 1 and 2. The increase in the prevalence of sexual behaviors was largely driven by responses from men. Women reported smaller increases in physical and virtual sexual behaviors between Wave 1 and 2. For participants in a steady relationship in both waves, there was an increase in the proportion whose scores indicated poor relationship quality between Wave 1 and Wave 2.

Studies conducted in the early months of the pandemic have pointed to declines in partnered sexual behavior in multiple countries, including the UK (Firkey et al., 2021; Ko et al., 2020; Lehmiller et al., 2021; McKay et al., 2023; Sanchez et al., 2020; Wignall et al., 2021). Cross-sectional analyses of the Natsal-COVID Wave 1 survey showed that men and women were more likely to perceive declines than increases in partnered sexual activities in the first four months of the pandemic, compared to before the lockdown (Mercer et al., 2022). Our study suggests that these early declines in frequency of partnered sexual activity did not persist twelve months into the pandemic, despite new rounds of lockdowns occurring in this period. In contrast to our results, a longitudinal study of sexual behavior among a convenience sample of US men who have sex with men observed a decrease in the number of sexual partners and opportunities to have sex in the early weeks of the pandemic, which was sustained until December 2020 (Mann et al., 2023). However, a different longitudinal study of men who have sex with men in Australia found gradual increases in sexual behavior with non-committed relationship partners over the pandemic, with localized differences associated with local lockdown restrictions (Storer et al., 2023). The results of our study suggests that the longer-term pandemic effects might differ by population and country context.

Our study suggests that after the initial phase of the pandemic, behavior reverted to pre-pandemic levels. There are several plausible mechanisms here. Restrictions changed over time, allowing adults to resume certain behaviors later in the pandemic. For example, between 13th June 2020 and 6^th^ July 2020, restrictions in Britain were eased to allow single adults who lived alone to have unrestricted mixing with a pre-designated, additional household. This permitted some non-cohabiting couples to resume partnered sexual activities whilst still adhering to pandemic restrictions. In addition, compliance with social restrictions declined over time for some in the UK (Wright et al., 2022), and so meeting of non-cohabiting sexual partners may have become more prevalent, despite restrictions.

Studies of solo sexual behaviors, conducted in the early months of the pandemic have consistently reported increases in the prevalence and/or frequency of masturbation and pornography use compared to pre-pandemic levels (Cascalheira et al., 2021; Hensel et al., 2023; Lau et al., 2021; Melca et al., 2021; Zattoni et al., 2020). Natsal-COVID Wave 1 data suggested that most participants reported no change in the frequency of masturbation or pornography use. However, amongst participants who did report a change, increases in frequency were more common than decreases (Mercer et al., 2022). Our study suggests that increases in the prevalence and frequency of masturbation and pornography use continued through the first year of the pandemic. It is not yet possible to say whether the observed increase in these solo sexual behaviors was a temporary response to pandemic restrictions experienced in both survey waves or a trend that will be sustained into the post-pandemic period.

Our study found that the increase in prevalence of sexual activity at Wave 2, compared with Wave 1, was moderated by gender. Both physical and virtual sexual behaviors showed greater increases in prevalence amongst men than women. Researchers have suggested that women’s sexual desire and function was negatively affected in the early stages of the pandemic (Hessami et al., 2022; Khoo et al., 2021) and that women’s sexual desire may have been more affected by the pandemic than men’s (Wignall et al., 2021). Our results suggest that this gender discrepancy in the effects of COVID on sexual activity and desire may have persisted across the first year of the pandemic. This finding is consistent with evidence that women experienced greater levels of stress and psychological distress than men during this time (Kowal et al., 2020; Moreno-Agostino et al., 2023).

Previous analyses have described a variety of changes to romantic partnerships in the first few months of the pandemic. Some studies reported reductions in perceived relationship quality (Balzarini et al., 2022; Ogan et al., 2021; Rodríguez-Domínguez et al., 2022; Schmid et al., 2021), but others reported neutral or positive impacts on relationship satisfaction (Holmberg et al., 2022; Williamson, 2020). Our results suggest that COVID-19 placed a continued strain on many relationships, with an increase in the proportion of participants experiencing less satisfying relationships. However, there was also movement from low quality relationships into higher quality, suggesting some variation in the way that relationships fared following the initial shock of the early pandemic. This finding is consistent with previous longitudinal research on relationship quality after trauma, which has found that periods of stress can cause a deterioration in relationship quality (Bodenmann, 1997; Neff & Karney, 2017; Rauer et al., 2008), but can also bond relationships together (Linley & Joseph, 2004). Natsal-COVID Wave 1 data suggested that younger participants were more likely to perceive both positive and negative changes in relationship quality after the first four months of UK lockdown (Mitchell et al., 2022). The longitudinal data similarly suggest that younger participants may have been more likely to experience declines in relationship quality after 1 year in lockdown then older participants, although small sample sizes meant that evidence for an association with age was not strong.

The Natsal-COVID study is the largest, quasi-representative national survey exploring the impact of the pandemic on sexual behaviors and lifestyles in Britain. The current study extends research conducted during the initial lockdown months to explore whether changes in sexual behavior and relationship quality were sustained one year into the pandemic. However, unlike other Natsal surveys, pandemic restrictions made it necessary to recruit participants from a web panel. Although the sample was weighted for gender, age, region, social grade, ethnicity, and sexual identity, those with time and/or inclination to participate in online surveys may not be representative of the UK population in their pandemic experiences (Holmberg et al., 2022). Volunteer web panels have been shown to provide biased prevalence estimates compared to a probability sample of the UK population, a limitation that is only partially mitigated by the use of quotas (Erens et al., 2014). The weighted Wave 1 and Wave 2 samples have previously been shown to over-represent non-heterosexual identities and under-represent married participants, compared to national population data (in part due to the exclusion of adults over 59 years) (Dema et al., 2022). The weighted longitudinal sample also underrepresented younger age groups compared to the full Wave 1 sample (Dema et al., 2022).

Sexual identity and gender identity are both important factors that can shape a participant’s sexual behavior and experience of relationships and may moderate how these experiences changed during the pandemic (Wignall et al., 2021). An important limitation of the study is that it was not possible to explore differences in experience for certain groups, such as the transgender community, due to small numbers. The sample size also precluded analyses restricted by region, which may be another moderating factor given that pandemic restrictions varied regionally. Previous research using the Natsal COVID Wave 1 results have suggested that sexual identity was not a risk factor for perceived decline in relationship quality during the first four months of the pandemic, but that living in a rural area was associated with perceived declines amongst men (Mitchell et al., 2022).

Relationship quality scores were dichotomized in the analysis, which loses information on the amplitude of relationship quality change compared to an analysis using the continuous relationship quality score. The decision to dichotomize was made to facilitate comparison between an analysis by Mitchell et al. (2022) exploring perceived changes in relationship quality over the initial four months of the pandemic restrictions using the same metric, and this study, which explored changes in relationship quality later into the lockdown period.

Although the study’s longitudinal analysis is a key strength, participants were only surveyed twice and so interpretation of changes should be made with caution. Our findings might be influenced by events particular to the timing of data collection, rather than representing smooth trends over the eight months between survey waves. The nature of the on/off cycles of lockdowns between the two surveys makes it difficult to disentangle shock/easing effects, cumulative/repeated effects, or lasting/shaping effects from the observed changes. The detected differences may also partly reflect established seasonal differences in wellbeing (Daly et al., 2022; Schlager et al., 1993); Wave 1 was conducted during UK summer months, while Wave 2 was conducted as winter transitioned to spring.

The lack of pre-pandemic baseline data makes it challenging to determine whether the changes observed over our study period represent a recovery in sexual behavior, compared to before the COVID-19 pandemic, or part of a longer-term trend. Previous comparisons between sexual health measures in Natsal survey data and national sexual health service surveillance data suggest that the changes in sexual health seen during the pandemic differed from secular trends (Dema et al., 2022; Mitchell et al., 2023); however, we lack such surveillance data for sexual behavior. Comparisons between Natsal-3, a cross-sectional nationally representative British probability sample survey undertaken in 2010–2012, and Natsal-COVID data, have suggested a decrease in frequency of sexual behaviors in the Natsal-COVID data (Dema et al., 2022; Mitchell et al., 2023). However, it is challenging to distinguish to what extent these changes are related to the pandemic, secular trends over the past decade, or other factors. The restrictions introduced during the COVID-19 pandemic caused an upheaval to romantic relationships with implications for the population’s sexual wellbeing and relationship quality (Mitchell et al., 2022). One year after the first social restrictions were introduced, we observed an increase in the prevalence of a broad range of sexual behaviors, compared to the first weeks of the pandemic period, suggesting some recovery in both desire and intimacy after a decline which occurred in the initial weeks of the pandemic. However, we found a slight increase in the proportion of participants reporting low relationship quality, suggesting that sustained pressure of the pandemic may have taken a toll on romantic relationships. This study fills a gap in our knowledge about the longer-term impact of the pandemic on sexual behavior and relationship health. Understanding the longer-term impact of the pandemic on sexual and relational health has implications for predicting demand for sexual health services during similar emergencies, and for understanding the broader health and mental health toll of the pandemic. Future studies are needed to disentangle whether the changes we observed, particularly the increased prevalence of solo activities like masturbation and pornography use, have persisted after the first year of the pandemic, reflecting a permanent change in sexual behavior, or whether they were temporary responses to social restrictions.

## Supplementary Material

Supplemental Material

## Data Availability

The data are available from the UK Data Archive: https://doi.org/10.5255/UKDA-SN-8865-2
