# Supplementary material for: Longitudinal Assessment of Sexual Behavior and Relationship Quality During the First Year of the COVID-19 Pandemic in Britain: Findings from a Longitudinal Population Survey (Natsal-COVID)
Source: J Sex Res. 2025 Jan 15:1–11. Online ahead of print. doi: 10.1080/00224499.2024.2432000 (PMC12291618; doi:10.1080/00224499.2024.2432000)
Supplement: Supplemental Material [file HJSR_A_2432000_SM0289.zip › HJSR_A_2432000_Supplementary Material.docx]

**Natsal COVID Longitudinal Analysis**

**Supplementary Material**

**Supplementary Table 1**

*Change in Prevalence of Sexual Behaviors from Natsal-COVID-1 to Natsal-COVID-2, by Gender^1^ at Wave 1*

| Sexual Behavior | Women (including trans women) aOR  (95% CI) | Men (including trans men) aOR  (95% CI) | *p* |
| --- | --- | --- | --- |
| **Any sexual activity** | 1.25 (1.03–1.52) | 2.28 (1.37–3.79) | .031 |
| Any physical sexual activity | 1.16 (0.94–1.44) | 2.20 (1.35–3.58) | .019 |
| Any partnered sex | 1.05 (0.86–1.28) | 1.09 (0.86–1.37) | .831 |
| Vaginal, oral, and/or anal sex | 1.00 (0.82–1.21) | 1.13 (0.90–1.41) | .409 |
| Other genital contact | 1.36 (1.13–1.64) | 1.21 (0.99–1.48) | .413 |
| Masturbation | 1.52 (1.34–1.73) | 1.56 (1.27–1.90) | .861 |
| Using sex toys | 1.38 (1.21–1.58) | 1.28 (1.07–1.53) | .519 |
| **Any virtual sexual activity** | 1.08 (0.93–1.25) | 1.34 (1.13–1.59) | .059 |
| Any excluding pornography use | 0.95 (0.79–1.13) | 0.98 (0.80–1.20) | .815 |
| Looking at pornography | 1.16 (1.00–1.36) | 1.22 (0.99–1.50) | .730 |
| Messaging via dating apps/online | 0.82 (0.66–1.02) | 0.93 (0.74–1.15) | .450 |
| Sexting | 1.07 (0.86–1.34) | 0.92 (0.73–1.16) | .367 |
| Using video or voice calls | 1.07 (0.80–1.41) | 1.14 (0.89–1.47) | .717 |
| Paying for online sexual services | 0.11 (0.67–1.84) | 0.77 (0.49–1.19) | .282 |

^1^ Under the Natsal-COVID approach to gender, data reported for men includes trans men and data reported for women includes trans women. Participants identifying their gender “in another way” are excluded from analyses stratified by gender, given the small sample size, but are included in analyses that are not stratified by gender.

**Supplementary Table 2**

*Change in Prevalence of Sexual Behaviors from Natsal-COVID-1 to Natsal-COVID-2, by Age at Wave 1*

| Sexual Behavior | Aged 17–34 aOR (95% CI) | Aged 35–59 aOR (95% CI) | *p* |
| --- | --- | --- | --- |
| **Any sexual activity** | 1.45 (0.86–2.47) | 1.51 (1.24–1.83) | .900 |
| Any physical sexual activity | 1.04 (0.64–1.69) | 1.60 (1.31–1.94) | .106 |
| Any partnered sex | 1.11 (0.82–1.52) | 1.04 (0.90–1.21) | . 706 |
| Vaginal, oral, and/or anal sex | 1.12 (0.83–1.52) | 1.02 (0.89–1.18) | .585 |
| Other genital contact | 1.28 (0.99–1.66) | 1.27 (1.10–1.47) | .967 |
| Masturbation | 1.33 (1.08–1.63) | 1.65 (1.46–1.88) | .072 |
| Using sex toys | 1.39 (1.16–1.66) | 1.28 (1.12–1.46) | .472 |
| **Any virtual sexual activity** | 1.15 (0.95–1.39) | 1.22 (1.07–1.39) | .611 |
| Any excluding pornography use | 0.89 (0.74–1.08) | 1.04 (0.87–1.26) | .257 |
| Looking at pornography | 1.12 (0.86–1.45) | 1.24 (1.09–1.41) | .480 |
| Messaging via dating apps/online | 0.85 (0.69–1.04) | 0.92 (0.73–1.16) | .617 |
| Sexting | 0.97 (0.79–1.20) | 1.00 (0.78–1.27) | .868 |
| Using video or voice calls | 1.09 (0.85–1.40) | 1.13 (0.86–1.48) | .862 |
| Paying for online sexual services | 0.73 (0.46–1.14) | 1.19 (0.74–1.90) | .136 |

**Supplementary Table 3**

*Change in Prevalence of Sexual Behaviors from Natsal-COVID-1 to Natsal-COVID-2, by Relationship Status at Wave 1*

| Sexual Behavior | In steady relationship aOR  (95% CI) | Not in steady relationship aOR  (95% CI) | *p* |
| --- | --- | --- | --- |
| **Any sexual activity** | 1.34 (0.99–1.81) | 1.66 (1.28–2.17) | .289 |
| Any physical sexual activity | 1.14 (0.84–1.56) | 1.69 (1.29–2.23) | .060 |
| Any partnered sex | 0.99 (0.82–1.19) | 1.19 (0.93–1.54) | .242 |
| Vaginal, oral, and/or anal sex | 0.93 (0.78–1.11) | 1.30 (1.00–1.70) | .036 |
| Other genital contact | 1.33 (1.12–1.57) | 1.20 (0.94–1.52) | .487 |
| Masturbation | 1.49 (1.30–1.71) | 1.65 (1.35–2.00) | .413 |
| Using sex toys | 1.32 (1.15–1.51) | 1.37 (1.13–1.65) | .758 |
| **Any virtual sexual activity** | 1.19 (1.05–1.36) | 1.21 (0.98–1.49) | .927 |
| Any excluding pornography use | 0.98 (0.82–1.17) | 0.94 (0.75–1.16) | .749 |
| Looking at pornography | 1.21 (1.07–1.38) | 1.15 (0.84–1.57) | .755 |
| Messaging via dating apps/online | 0.92 (0.74–1.14) | 0.83 (0.66–1.05) | .523 |
| Sexting | 0.96 (0.77–1.19) | 1.03 (0.81–1.32) | .649 |
| Using video or voice calls | 0.95 (0.74–1.22) | 1.47 (1.08–1.99) | .030 |
| Paying for online sexual services | 0.75 (0.55–1.03) | 1.04 (0.40–2.72) | .527 |

**Supplementary Table 4**

*Percent change in Prevalence of Sexual Behaviours from Natsal-COVID-1 to Natsal-COVID-2, by Cohabitation Status among Those in Steady Relationships Using Cross-Sectional Data*

| Sexual Behaviour | In steady non-cohabiting relationship (%) | In steady cohabiting relationship (%) |
| --- | --- | --- |
| **Any sexual activity** | +1.0 | +1.6 |
| Any physical sexual activity | +1.7 | +1.4 |
| Any partnered sex | +1.9 | +0.1 |
| Vaginal, oral, and/or anal sex | +2.2 | -0.4 |
| Other genital contact | +7.3 | +3.8 |
| Masturbation | +9.5 | +8.5 |
| Using sex toys | +7.1 | +5.6 |
| **Any virtual sexual activity** | +4.9 | +4.4 |
| Any excluding pornography use | +0.8 | +1.0 |
| Looking at pornography | +8.6 | +4.2 |
| Messaging via dating apps/online | -6.5 | +0.8 |
| Sexting | +6.8 | +0.7 |
| Using video or voice calls | +1.7 | +0.1 |
| Paying for online sexual services | +2.6 | +0.6 |

**Supplementary Table 5**

*Comparison of the Characteristics of Participants Who Were Missing Data on the “Any Sexual Activity” Variable in Either Wave 1 or 2 versus Those Who Were Not Missing Sexual Behavior Data in Either Wave*

|  | Missing data  % (95% CI) | Not missing data  % (95% CI) |
| --- | --- | --- |
| **Age group** |  |  |
| 18–24 | 7.6 (3.3–16.5) | 11.4 (9.0–14.3) |
| 25–29 | 24.1 (15.5–35.6) | 18.5 (16.1–21.2) |
| 30–39 | 18.5 (12.3–27.0) | 24.7 (22.2–27.2) |
| 40–49 | 23.3 (17.0–31.0) | 23.0 (20.9–25.4) |
| 50–59 | 26.5 (20.0–34.3) | 22.5 (20.5–24.5) |
| **Gender** | | |
| Women (including trans women) | 58.7 (49.1–67.6) | 49.6 (46.7–52.6) |
| Men (including trans men) | 41.3 (32.4–51.0) | 50.2 (47.2–53.1) |
| In another way | 0.0 (—) | 0.2 (0.0–0.8) |
| **Relationship status** | | |
| Steady and cohabiting | 54.0 (44.2–63.6) | 61.4 (58.4–64.3) |
| Steady and not cohabiting | 3.6 (1.4–8.8) | 6.3 (5.0–7.8) |
| Single | 38.8 (29.6–48.9) | 27.7 (24.9–30.6) |
| Other relationship status | 3.5 (1.3–9.5) | 4.7 (3.6–6.1) |
| **Ethnicity** | | |
| White | 79.9 (70.0–87.1) | 86.3 (83.5–88.7) |
| Mixed/Multiple | 0.5 (0.2–2.2) | 2.1 (1.4–3.1) |
| Asian/Asian British | 14.0 (8.0–23.4) | 7.6 (5.7–10.0) |
| Black/African/Caribbean | 4.3 (1.5–11.6) | 3.3 (2.1–4.8) |
| Other | 1.3 (0.2–8.7) | 0.8 (0.3–2.2) |
| **Household** | | |
| Living with children <18 | 23.3 (16.1–32.4) | 32.2 (29.5–35.0) |
| **Social grade** | | |
| Upper middle/Middle class | 23.2 (16.3–32.0) | 20.5 (18.4–22.7) |
| Lower middle/Skilled working class | 51.0 (41.4–60.5) | 54.1 (51.2–57.0) |
| Working class/Lower subsistence | 25.8 (17.8–35.9) | 25.4 (22.8–28.2) |

**Supplementary Table 6**

*Prevalence of Sexual Behaviors in Previous Four Months, Natsal-COVID-1 versus Natsal-COVID-2, Using an Alternative Definition of Missing Data*

| Sexual Behavior | Wave 1 (%) | Wave 2 (%) | aOR (95% CI) | *p* |
| --- | --- | --- | --- | --- |
| Any sexual activity | 84.1 | 86.1 | 1.19 (1.01–1.40) | .041 |
| Any physical sexual activity | 82.2 | 85.0 | 1.25 (1.05–1.48) | .011 |
| Any partnered sex | 63.5 | 64.6 | 1.06 (0.91–1.23) | .432 |
| Any virtual sexual activity | 49.5 | 51.9 | 1.13 (1.02–1.25) | .022 |
| Any virtual excluding pornography use | 26.3 | 25.5 | 0.95 (0.83–1.09) | .466 |

*Note.* In the main analysis, aggregate sexual behavior variables are defined as missing if *any* component behaviors are missing responses and *no* component behaviors were performed. In this supplementary analysis, aggregate behaviors are defined as missing if *all* component behaviors are defined as missing.

**Supplementary Table 7**

*Change in the Prevalence of Low Relationship Quality Score between Natsal-COVID-1 versus Natsal-COVID-2*

| Sexual Behavior | Wave 1 (%) | Wave 2 (%) | aOR (95% CI) | *p* |
| --- | --- | --- | --- | --- |
| All participants | 23.9 | 26.9 | 1.28 (1.10-1.49) |  |
| Women | 24.1 | 32.9 | 1.47 (1.14-1.90) | .097 |
| Men | 24.2 | 26.8 | 1.13 (0.95-1.35) |  |
| 17–34-year-olds | 22.9 | 28.5 | 1.34 (1.00-1.80) | .686 |
| 35–59-year-olds | 24.9 | 29.3 | 1.25 (1.05-1.49) |  |
| Cohabiting at Wave 1 | 24.8 | 30.3 | 1.27 (1.09-1.49) | .780 |
| Not cohabiting at Wave 1 | 17.8 | 23.4 | 1.40 (0.72-2.73) |  |

*Note*. *P*-value represents the significance test for each interaction (i.e., by gender, by age group, by cohabitation status).

Under the Natsal-COVID approach to gender, data reported for men includes trans men and data reported for women includes trans women. Participants identifying their gender “in another way” are excluded from analyses stratified by gender, given the small sample size, but are included in analyses that are not stratified by gender.
