# Supplementary material for: Longitudinal Assessment of Sexual Behavior and Relationship Quality During the First Year of the COVID-19 Pandemic in Britain: Findings from a Longitudinal Population Survey (Natsal-COVID)
Source: J Sex Res. 2025 Jan 15:1–11. Online ahead of print. doi: 10.1080/00224499.2024.2432000 (PMC12291618; doi:10.1080/00224499.2024.2432000)
Supplement: Supplemental Material [file HJSR_A_2432000_SM0289.zip › HJSR_A_2432000_Supplementary Material_2.doc]

## Supplementary Material

# **Supplementary Table 1: Summary of survey measures and derivation of variables for analysis**

| Measure | Questions used to collect data for measure* | Description of how measure was derived and/ or analysed |
| --- | --- | --- |
| **Definition of sample** | | |
| Steady relationship | 1. Which statement best describes your **current** relationship status?   Response options:   A. Married / in a civil partnership and living together B. Married / in a civil partnership and **not** living together C. In a steady relationship and living together D. In a steady relationship and **not** living together E. In a new relationship F. In a casual relationship G. Single H. At the end of a relationship (e.g. separating) I. In more than one type of relationship J. Other K. Prefer not to say | Survey participants were defined as being in a steady relationship if they reported A, B, C or D as their current relationship status and their relationship status before lockdown. |
| **Relationship quality** | | |
| Relationship quality since lockdown | Thinking about your relationship **since lockdown** (23 March 2020), please read the statements below carefully and tell us to what extent you agree or disagree. *(i.e., current relationship quality)*  Questions:   1. I feel **supported** by my partner 2. My partner and I regularly **argue** 3. I feel a **strong connection** with my partner 4. I can **confide** in my partner about virtually anything 5. I am **worried** that our relationship might end 6. I feel **happy** with my relationship   Response options:  Not at all true; A little true; Somewhat true; Mostly true; Completely true; Prefer not to say | Derived variable: A/B= not true; C=somewhat true; D/E= true  Analysis: Proportion reporting each of three categories (more true/no change/less true) by age. Significance testing for variation by age.  Data are not disaggregated by gender because preliminary analysis suggested no significant differences by gender. |

# **Supplementary material 2: Method for scoring overall relationship quality and change in relationship quality compared with before lockdown began.**

**Exploratory factor analysis**

We conducted factor analysis for the question bank on relationship quality since lockdown (6 items) and change in relationship quality (perceived change on same 6 items). Cronbach alphas were 0.81, and 0.84 respectively.

| Factor | Factor loadings | Unique variances | Cronbach alpha |
| --- | --- | --- | --- |
| Current relationship quality (since lockdown) | | | 0.81 |
| Support | 0·832 | 0·308 |  |
| Argue | 0·544 | 0·704 |  |
| Connection | 0·849 | 0·279 |  |
| Confide | 0·832 | 0·307 |  |
| Worry | 0·648 | 0·580 |  |
| Happy | 0·875 | 0·234 |  |
| Change in relationship quality (compared with before lockdown) | | | 0.84 |
| Support | 0·7464 | 0·4429 |  |
| Argue | 0·4731 | 0·7762 |  |
| Connection | 0·8274 | 0·3155 |  |
| Confide | 0·8028 | 0·3555 |  |
| Worry | 0·6409 | 0·5892 |  |
| Happy | 0·8240 | 0·3211 |  |
|  |  |  |  |

**Table i: Factor loadings for items in each question bank.**

**Creating combined scores for relationship quality since lockdown**

Response options were scored 1 to 5 (1 for not at all true and 5 for completely true; negative statements reverse coded) and each score was multiplied by the factor loading for that item. The distribution of scores for weighted relationship quality scores (see ) were used to identify cut-offs. These cut-offs were first identified as empirical thresholds using the histograms and then cross-referenced and compared with percentiles of score distribution values. For relationship quality, the observed cut-off of 17 corresponded to approximately being below the 25^th^ percentile. As a result, for relationship quality since lockdown, a weighted score below <17 was categorised as ‘lower quality’ (n=917 in baseline sample) and a score greater than or equal to 17 was categorised as ‘higher quality’ (n=3,003 in baseline sample).

**Figure i** Relationship quality score distribution weighted by factor loadings.
